# Novel trends of genome evolution in highly complex tropical sponge microbiomes

**DOI:** 10.1186/s40168-022-01359-z

**Published:** 2022-10-04

**Authors:** Joseph B. Kelly, David E. Carlson, Jun Siong Low, Robert W. Thacker

**Affiliations:** 1 Aquatic Ecology and Evolution, Limnological Institute University Konstanz, Konstanz, Germany; 2 Department of Ecology and Evolution, Stony Brook University, Stony Brook, NY USA; 3 Institute of Microbiology,ETH Zürich, Zürich, Switzerland; 4 Institute for Research in Biomedicine, Università della Svizzera Italiana, Bellinzona, Switzerland; 5 Department of Immunobiology, Yale University School of Medicine, New Haven, CT USA; 6 Smithsonian Tropical Research Institute, Box 0843-03092, Balboa, Panama City, Republic of Panama

**Keywords:** Sterols, Metagenomics, Microbiome, Sponge, *Ircinia*, Marine, Tropical

## Abstract

**Background:**

Tropical members of the sponge genus *Ircinia* possess highly complex microbiomes that perform a broad spectrum of chemical processes that influence host fitness. Despite the pervasive role of microbiomes in *Ircinia* biology, it is still unknown how they remain in stable association across tropical species. To address this question, we performed a comparative analysis of the microbiomes of 11 *Ircinia* species using whole-metagenomic shotgun sequencing data to investigate three aspects of bacterial symbiont genomes—the redundancy in metabolic pathways across taxa, the evolution of genes involved in pathogenesis, and the nature of selection acting on genes relevant to secondary metabolism.

**Results:**

A total of 424 new, high-quality bacterial metagenome-assembled genomes (MAGs) were produced for 10 Caribbean *Ircinia* species, which were evaluated alongside 113 publicly available MAGs sourced from the Pacific species *Ircinia ramosa*. Evidence of redundancy was discovered in that the core genes of several primary metabolic pathways could be found in the genomes of multiple bacterial taxa. Across hosts, the metagenomes were depleted in genes relevant to pathogenicity and enriched in eukaryotic-like proteins (ELPs) that likely mimic the hosts’ molecular patterning. Finally, clusters of steroid biosynthesis genes (CSGs), which appear to be under purifying selection and undergo horizontal gene transfer, were found to be a defining feature of *Ircinia* metagenomes.

**Conclusions:**

These results illustrate patterns of genome evolution within highly complex microbiomes that illuminate how associations with hosts are maintained. The metabolic redundancy within the microbiomes could help buffer the hosts from changes in the ambient chemical and physical regimes and from fluctuations in the population sizes of the individual microbial strains that make up the microbiome. Additionally, the enrichment of ELPs and depletion of LPS and cellular motility genes provide a model for how alternative strategies to virulence can evolve in microbiomes undergoing mixed-mode transmission that do not ultimately result in higher levels of damage (i.e., pathogenicity) to the host. Our last set of results provides evidence that sterol biosynthesis in *Ircinia*-associated bacteria is widespread and that these molecules are important for the survival of bacteria in highly complex *Ircinia* microbiomes.

Video Abstract

**Supplementary Information:**

The online version contains supplementary material available at 10.1186/s40168-022-01359-z.

## Background

Sponges (phylum Porifera) are exceptional examples of host-microbial associations in the aquatic environment. In some species, microbes can comprise up to 40% of the sponge biomass [1, 2], and the richness of operational taxonomic units (OTUs) in the microbiome can number in the thousands [3]. One such genus that has a particularly noteworthy relationship with symbiotic microbes is *Ircinia.* Tropical species of *Ircinia* contain abundant populations of Cyanobacteria and hundreds to thousands of other microbial species belonging to dozens of phyla [3–7]. Experiments measuring host growth in light vs. dark conditions in other genera have demonstrated that “*Candidatus* Synechococcus spongiarum” (Cyanobacteria) engages in a nutritional symbiosis with sponges [8, 9], a phenomenon that is likely present in *Ircinia* given the abundance of chlorophyll *a* in many species and the presence of “*Ca*. S. spongiarum” as evidenced by 16S rRNA barcoding [10], although it should be noted that some cyanobacterial symbionts of sponges, such as “*Ca.* Synechococcus feldmannii,” do not contribute to the host’s carbon budget [11]. The microbial symbionts in *Ircinia* also influence the host’s biology by contributing to nitrogen, carbon, and phosphorous cycling [12, 13] and by underlying the production of biologically active secondary metabolites [14, 15 ].

Recent research on tropical *Ircinia* has focused on the evolutionary impacts of microbial symbioses by investigating the host transcriptomes [4], 16S rRNA metabarcoding of microbiomes [4 , 5], microbial metagenomes [16], and host genomes using microsatellites [5] and restriction site-associated DNA sequencing (RADseq) [4 ]. In Caribbean *Ircinia*, two studies discovered that microbiome dissimilarities scale positively with host genetic distances, suggesting a potential role of the microbiome in ecological divergence among the host species [4, 5]. Additionally, an analysis mapping RADseq loci that were *F*_*ST*_ outliers to an *Ircinia* transcriptome assembly inferred selection in host genes that are part of the lipopolysaccharide (LPS)-induced Ras-Raf-MEK-ERK (MAPK/ERK) pathway and selection in other genes potentially relevant to the regulation of host-microbiome interactions [4]. Given the presence of this pathway, one possibility is that the bacteria in *Ircinia* have lost LPS to avoid digestion, a hypothesis corroborated by the depletion of LPS-related genes in “*Ca.* S. spongiarum,” which is exposed to the archaeocytes of the sponge [17, 18 ].

A previous study of the Pacific species *Ircinia ramosa* used a metagenomic approach to examine how microbes evolve within this host [16]. This study found redundancy in several primary metabolic pathways including nitrogen, sulfur, and carbon metabolism, which could result in a portfolio type effect [19] that buffers the core metabolic functioning from taxonomic fluctuations in the microbiome. The *I. ramosa* metagenome also contained ankyrin (*ANK*) and *WD40* repeat-bearing eukaryotic-like proteins (ELPs), which are generally accepted as aiding prokaryotes in evading the host’s immune apparatuses by mimicking the host sponge’s molecular patterning [20]. The present study sought to expand our understanding of genome evolution in the bacterial symbionts of tropical *Ircinia* by performing a whole-shotgun metagenomic survey of ten Caribbean species that were previously investigated for genome-wide patterns of evolution in the sponge hosts [4], six of which are newly described species [21]. Given the high level of complexity within the microbiomes of tropical *Ircinia*, the evidence of selection in host genes that are involved in immune responses to microbial pathogens, the abundance of bioactive compounds in *Ircinia*, and their potential to influence host-microbe interactions, we hypothesized that within the metagenomes of *Ircinia* we would find further evidence for metabolic redundancy among multiple bacterial taxa, depletion of LPS biosynthesis and other genes related to pathogenicity, and purifying selection in genes involved in the biosynthesis of secondary metabolites.

## Methods

### Sample collections, DNA extractions, and sequencing

New metagenome-assembled genomes (MAGs) were produced for this study from two specimens of each of ten *Ircinia* species, resulting in a sample size of 20. In Bocas del Toro, Panama, specimens of *Ircinia radix* were collected from prop roots of the mangrove hammock at Inner Solarte (latitude, longitude: 9.3058, − 82.1732), individuals of *Ircinia laeviconulosa* and *I. bocatorensis* from seagrass beds near the Smithsonian Tropical Research Institute’s Bocas del Toro Research Station (9.3517, − 82.2590), and individuals of *Ircinia lowi* from patch reefs at Punta Caracol (9.3771, − 82.3023). In Florida, specimens of *Ircinia campana* and *Ircinia* cf. *reteplana* Topsent, 1923, were collected from a seagrass bed near the MOTE Marine Laboratory and Aquarium’s Elizabeth Moore International Center for Coral Reef Research & Restoration (24.6609, − 81.4563). In Belize, specimens of *Ircinia vansoesti* were collected from the prop roots of mangrove hammocks near the Blue Ground site (16.8083, − 88.1496), specimens of *Ircinia strobilina* and *Ircinia ruetzleri* were collected from the coral patch reefs of Blue Ground (16.8010, − 88.1461), and specimens of *Ircinia felix* were collected from the forereef of Carrie Bow Cay (16.8042, − 88.0796). Thumb-size fragments were cut from the outer region of the sponges’ bodies and stored in 90% EtOH that was replaced at the 24- and 48-h time points to ensure thorough inundation and preservation of the microbial cells. The exterior surfaces of the sponges were free of overgrowth with the exception of *I. vansoesti*, from which the loosely attached overgrowth easily sloughed away upon sample collection. Photos and descriptions of the growth forms and maps of the sampling locations can be found in [4]. The specimens used in the current study are present in the set of specimens used in [4].

DNA was extracted from a 0.5 cm × 0.5 cm × 0.5 cm fragment cut from the outermost region of each specimen using the Molzyme Ultra-Deep Microbiome Prep DNA isolation kit according to the manufacturer’s instructions. This kit enriches prokaryotic DNA by first lysing eukaryotic cells and destroying the then free-floating eukaryotic DNA and subsequently lysing the prokaryotic cells and isolating the DNA therein [22, 23]. The absence of host DNA in our samples was evaluated by amplifying the Molzyme DNA isolations with *Ircinia*-specific cytochrome oxidase c subunit 1 (CO1) primers cox1.IrcF (GAT AAT GCG GYT CGA GTT GK) and cox1.IrcR (CTA CCG GAT CAA AGA AAG AAG TRT) in HotStarTaq Master Mix (QIAGEN). PCR amplifications were conducted using cycles of 95 °C for 15 min; 35 cycles at 94 °C for 1 min, 62 °C for 1 min, and 72 °C for 1.5 min; and a 10-min-long final elongation step. To provide control isolation with host DNA, PCR amplifications were repeated for the same samples using bulk-tissue DNA isolations produced using the Wizard Genomic DNA Purification kit (Promega). Previous 16S rRNA metabarcoding of these same samples demonstrated that they contain only trace relative abundances of environmental seawater bacteria [4]; thus, contaminating DNA from the seawater microbial community is expected to be negligible. Whole-metagenome shotgun sequencing was performed for the Molzyme DNA extractions after confirming the absence of a CO1 amplification band at the Yale Center for Genome Analysis on a NovaSeq6000 with a target of 30 million 2 × 150 reads per specimen.

### Metagenomic assembly, binning, dereplication, and annotation

Reads were filtered and trimmed using FastP [24] with default parameters. Bbsplit.sh [25] was used to remove PhiX control reads and reads mapping to the *Mus musculus* and *Homo sapiens* genomes, which was performed as a precautionary step since the DNA libraries were prepared in a facility that routinely handles vertebrate model organisms. Metagenomic contigs were assembled independently for each specimen using Megahit v1.2.9 [26 ] and subsequently binned using MetaBAT 2 v2:2.15 [27] with default parameters. Contaminating contigs were identified on the basis of being outliers with regard to genomic content (i.e., %GC and tetranucleotide content) and removed from the bins using RefineM v0.0.25 [28]. The qualities of the refined bins were assessed using CheckM v1.1.2 [29 ]; only bins with quality scores equal to or higher than a cutoff score of 40, calculated as genome completeness—5× contamination [28]—and which had at least 85% genome completeness were retained for downstream analysis. MAGs were then dereplicated using dRep v2.5.2 [30] within each host species using a cutoff of 96.5% average nucleotide identity, an empirically derived estimate of maximum prokaryotic intraspecific genomic divergence [31]. Protein predictions and KEGG Orthology (KO) annotations (http://www.kegg.jp/kegg/) were performed on the MAGs using EnrichM v0.5.0 [32]. Pfam annotations were performed on the protein domains using Interproscan v5.39-77 [33]. For proteins that contained duplicated identical domain annotations, only one domain was retained to avoid double-counting proteins. The relative abundances of each MAG per host specimen were evaluated with CoverM v0.4.0 (https://github.com/wwood/CoverM) using the concatenated dereplicated Caribbean *Ircinia* MAG dataset. We chose this scheme as previous research using 16S rRNA metabarcoding demonstrated a high degree of symbiont sharing, although at varying relative abundances [4].

### Processing of publicly available *I. ramosa* symbiont and Tara oceans MAGs

Publicly available *I. ramosa* symbiont MAGs (NCBI BioProject ID PRJNA555144) and MAGs produced from pelagic samples collected during the Tara Oceans Expedition that correspond to North Atlantic and South Pacific oceanic provinces (NCBI BioProject ID PRJNA391943) [34] were downloaded from NCBI. The quality filtering, dereplication, and annotation steps were performed as outlined above. Taxonomy was assigned to the MAGs, and a whole-genome phylogenetic tree was produced for the *Ircinia* MAGs using GTDB-Tk v1.0.2 [35].

### Enrichment analysis

We compared MAGs sourced from each *Ircinia* host species to Tara Oceans MAGs from the oceanic province that encompassed the collection sites of the sponges (North Atlantic for the ten Caribbean *Ircinia* species and South Pacific for *I. ramosa*). Pairwise permutational *t*-tests were run for 10,000 iterations that compared the average abundance (e.g., copy number) of a feature in the MAGs belonging to a given *Ircinia* species against the Tara Oceans datasets. To factor in differences in genome size, we normalized feature counts by dividing them by the total number of predicted proteins prior to the *t*-tests. *p*-values were corrected using the false discovery rate adjustment of Benjamini-Hochberg [36] using the total number of features within an annotation category (either KO or Pfam). Given the high number of multiple comparisons, we further decided to assign a conservative significance cutoff of *p* < 0.01. Additionally, we omitted the features that were present in less than 10% of both the *Ircinia* and Tara Oceans MAGs. The enrichment analysis pipeline was also performed at the phylum level (or class level, in the case of Proteobacteria) comparing Tara Oceans MAGs against *Ircinia* MAGs. To ensure sufficient sample sizes for the analysis, this analysis grouped samples across sources and required a minimum of ten MAGs belonging to each life history category.

### Inference of selection, homology search, and phylogenetic analysis in CSGs

We subjected the most common cluster of steroid genes (CSGs) to variant analysis. In the order that they appear in the CSGs, these genes are delta14-sterol reductase [EC:1.3.1.70] (*TM7SF2/ERG24*), sterol 14alpha-demethylase [EC:1.14.14.154] (*CYP51*), and lanosterol synthase [EC:5.4.99.7] (*LSS*/*ERG7*). We restricted the analysis to the ten Caribbean *Ircinia* species as they all shared the same DNA extraction, library prep, sequencing, and MAG refinement pipeline. Preparation for this dataset differed from the enrichment analysis dataset in that the MAGs underwent dereplication across the ten host species instead of within each host species. This dereplication scheme produced fewer MAGs, which reduced computational burden, a necessary consideration to keep the wall time of the analysis and storage space of the intermediate files within feasibility.

Variants were called by mapping the cleaned and filtered reads for each metagenomic sample to each MAG using BWA MEM v0.7.17 [37]. Alignments were converted to binary format and sorted by position with Samtools v1.9 [38]. PCR and optical duplicate reads were removed from the alignments using the “MarkDuplicates” utility in Picard v2.22.2 (http://broadinstitute.github.io/picard/). Variants were called using the “mpileup” and “call” functions in BCFtools v1.9 [39]. The resultant VCF files were also filtered using BCFtools to remove variants that had QUAL scores less than 20 or if read depth was anomalously high [40]. This pipeline was run for each replicated MAG using the Snakemake workflow management system v.5.13.0 [41]. Nucleotide diversity was measured for the three core genes within the CSGs as a function of average variable sites per nucleotide and was compared against the genome-wide average of nucleotide diversity for all coding sequences, less the three CSG genes, using *t*-tests.

The selection was also inferred in the three core genes contained in the CSGs by comparing the ratio of nonsynonymous (dN) to synonymous (dS) substitutions. Codon-aware alignments were constructed of each of the three sets of genes using Mafft v7.149 [42], implemented in translatorx v1.1 [43]. Maximum likelihood gene trees were inferred for each alignment using IQ-Tree v2.1.4 [44], with best-fitting substitution models chosen by ModelFinder [45] and branch support estimated using 1000 ultrafast bootstrap replicates [46]. Next, we used the CODEML program in PAML v4.10.0 [47] to estimate omega values (dN/dS) as a proxy of selection acting on each gene. CODEML was run using each alignment and gene tree with model 0 to infer a single estimate of omega for each gene. This pipeline was repeated for 1000 randomly chosen genes that were present in at least 10% of the *Ircinia*-sourced MAGs to provide a distribution of omega values for comparison. A quality filter was applied that required genes to have a tree-wide average ultrafast-bootstrap value of at least 70% to be retained for the final distribution.

To investigate the potential patterns of horizontal gene transmission (HGT), phylogenetic trees were produced via Bayesian inference for each of the three genes in the CSGs using the codon-aware alignments constructed for the aforementioned CODEML analysis. Prior to tree inference, the models of nucleotide evolution best fitting each alignment were selected using JModelTest v2.1.10, implemented in the CIPRES Science Gateway (www.phylo.org/portal2) [48]. Two parallel runs each with four chains were then run MrBayes v3.2.6 [49] until the average standard deviation of split frequencies fell below 0.01.

We performed a sequence homology search to investigate the presence of the CSGs in other bacterial genomes by querying sequences of the complete three-gene CSGs to NCBI’s nucleotide collection (nr) database (accessed 12 August 2021) using the online version of BLASTn [50] with default settings. The query sequences we chose for this analysis were selected to represent each host species and bacterial phylum of the MAGs in which the CSGs were present. Additionally, we searched for the CSGs in the publicly available MAGs of three non-*Ircinia* host species (*Aplysina aerophoba*, NCBI BioProject ID PRJNA326328 and PRJNA274222; *Spongia spongia*, PRJEB18465; and *Mycale hentscheli*, PRJNA603662) and the Mediterranean Tara Oceans MAGs (the oceanic province from which *A. aerophoba* samples were collected) by subjecting them to the same quality control (QC), dereplication (within species), and annotation pipeline as above.

## Results

### Metagenomic sequencing of ten Caribbean *Ircinia* species’ microbiomes

**Fig. 1:**
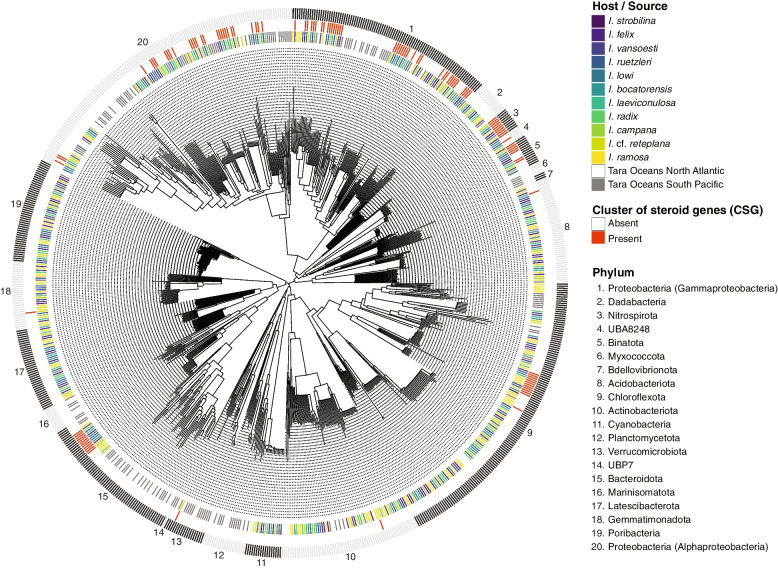
Whole-genome phylogenetic tree of MAGs sourced from bacterial symbionts of *Ircinia* spp. and North Atlantic and South Pacific Tara Oceans MAGs, produced using the MAG dataset that was dereplicated within each source using GTDB-Tk. The inner ring indicates the source of the MAGs, the middle ring denotes the presence or absence of CSGs, and the outer ring denotes the GTDB-Tk phylum-level taxonomic annotations. Tree plotting and annotation were performed with the R package ggtree v2.0.4 [51]

Whole-metagenome shotgun sequencing was performed on the microbiomes of ten Caribbean *Ircinia* species. Contigs binned into 895 MAGs (mean = 89.5 ± 30.8 [1 s.d.] per species), which were reduced to 426 MAGs upon dereplication and the CheckM-based QC steps (mean = 42.6 ± 17.1 [1 s.d.] per species) (Additional file 6: Table S1). The species with the fewest MAGs was *I. strobilina* (*n* = 16), and the species with the most MAGs was *I. ruetzleri* (*n* = 62). Only two archaeal MAGs were recovered; thus, we decided to only retain the remaining 424 bacterial MAGs for downstream analysis. The MAGs encompassed 14 bacterial phyla (Additional file 6: Table S1, Fig. 1). The most numerous phyla in the Caribbean *Ircinia* metagenomes in terms of MAG richness were Proteobacteria (68 Alphaproteobacteria and 49 Gammaproteobacteria), Chloroflexota (82 MAGs), and Poribacteria (40 MAGs). Additionally, a *Trichodesmium* (Cyanobacteria) that was initially present in the pre-QC *I.* cf. *reteplana* dataset was removed as it did not meet the 85% completeness threshold (jk18x7bins.61, quality = 77.0, completeness = 80.8%, contamination = 0.8%). Although the *Trichodesmium* and archaeal MAGs were not used in downstream analyses, we have included them in the NCBI BioProject.

On average, 57.5% ± 17.2 [1 s.d.] raw reads mapped back to the bacterial MAGs per host specimen during the CoverM analysis (Additional file 1: Fig. S1). 3.3% of MAGs were unique to only one host species whereas 77.4% of MAGs were found in at least half of the species (Additional file 2: Fig. S2A). 7.1% of MAGs were unique to only one of the three Caribbean locales, and 69.6% of MAGs were found across all three sites (Additional file 2: Fig. S2B). A PCA of the relative abundances of MAGs showed specimens clustering together by host species (Additional file 2: Fig. S2C). *Synechococcus* was by far the most abundant microbial genus in our dataset; on average, it comprised 21.6% ± 11.6 [1 s.d.] of the total microbiome composition per host specimen and was present in every host taxon except for *I. strobilina*. Multiple *Synechococcus* MAGs were found within a given specimen, although only a fraction of the *Synechococcus* MAGs were dominant in each host taxon with the exception of *I. strobilina*, which contained no *Synechococcus* (Additional file 7: Table S2).

### General comparison of *I. ramosa* to Caribbean *Ircinia* metagenomes

A total of 113 bacterial MAGs passed the quality filtering and dereplication pipeline for *I. ramosa* (Additional file 6: Table S1). Of the 14 phyla found in Caribbean *Ircinia*, two were absent in *I. ramosa*: Binatota and Verrucomicrobiota. Fourteen MAGs belonging to the class Binatia (phylum Binatota) were recovered in the Caribbean *Ircinia* dataset, with at least one MAG from this class found in each host species’ dataset except for *I. campana*, although the CoverM analysis revealed that members of this class were present in this host species at low abundances. A MAG (jk18x7bins.13) belonging to the order Opitutales (phylum Verrucomicrobiota) was found in the set of MAGs from *I.* cf. *reteplana*, and the CoverM analysis showed the additional presence of this MAG in *I. campana*, *I. ruetzleri*, and *I. felix*.

Similar to *I. ramosa*, a metabolic characterization of the Caribbean *Ircinia* MAGs showed redundancy across bacterial taxa in genes involved in nitrogen metabolism (PATH:ko00910), sulfur metabolism (PATH:ko00920), and the six autotrophic carbon cycling pathways of prokaryotes: the reductive citric acid cycle, the Wood-Ljungdahl pathway, the 3-hydroxypropionate bicycle, the 3-hydroxypropionate/4-hydroxybutyrate cycle, the Calvin-Benson-Bassham cycle, and the dicarboxylate/4-hydroxybutyrate cycle (Additional file 1: Fig. S1). However, nickel-CODH (cooS, acsA, K00198) was absent from all MAGs (Additional file 1: Fig. S1), concurrent with previous reports of this gene and, consequently, the Wood-Ljungdahl pathway being absent from sponge microbiomes [11]. Additionally, we discovered a pattern of redundancy within methane metabolism (PATH:ko00680).

### Enrichment patterns of tropical *Ircinia* metagenomes

To investigate the patterns of genomic enrichment in the symbiotic bacteria associated with tropical *Ircinia*, we compared the dataset of MAGs from the ten Caribbean *Ircinia* species and publicly available MAGs sourced from the Pacific species *I. ramosa* to MAGs assembled from samples collected during the Tara Oceans Expedition [34]. Since the Tara Oceans dataset was produced from DNA isolated from filtered oceanic seawater, we assume that these microbes are pelagic [34]. A similar analytical scheme has been adopted before to study the genomic enrichment patterns in *Porites lutea* microbial metagenomes [52]. The present analysis compared MAGs found within each host species to Tara Oceans MAGs from the oceanic province that encompassed the collection site of the host species and was performed within each species and independently for bacterial phyla with at least ten MAGs in each life history category. A gene was considered enriched if its average copy number was significantly greater in the sponge MAG dataset relative to the corresponding Tara Oceans dataset. Conversely, a gene was considered depleted in the *Ircinia* metagenomes if the average copy number was significantly lower relative to its average copy number in the Tara Oceans dataset.

Following quality filtering and dereplication, 101 bacterial MAGs (of the original 286) representing 12 phyla were recovered for the North Atlantic and 158 MAGs (of the original 474) representing 15 phyla were recovered from the South Pacific Tara Oceans datasets (Additional file 6: Table S1). A high degree of taxonomic overlap between the *Ircinia* and Tara Oceans MAGs was observed. 75.9% of the South Pacific MAGs belonged to the phyla found also in *I. ramosa*, and 85.6% of *I. ramosa* MAGs belonged to the phyla found in the South Pacific dataset. Likewise, 83.2% of the North Atlantic MAGs belonged to the phyla also present in the Caribbean *Ircinia* dataset, and 72.8% of the Caribbean *Ircinia* MAGs belonged to the phyla also found in the North Atlantic dataset. Four phyla contained at least ten free-living and ten sponge-associated MAGs (Actinobacteriota, 14 Tara Oceans and 51 *Ircinia* MAGs; Bacteroidota, 47 Tara Oceans and 18 *Ircinia* MAGs; Chloroflexota, 23 Tara Oceans and 115 *Ircinia* MAGs; and Proteobacteria). Proteobacteria was further split at the class level into Alphaproteobacteria (68 Tara Oceans and 73 *Ircinia* MAGs) and Gammaproteobacteria (38 Tara Oceans and 58 *Ircinia* MAGs).

**Fig. 2:**
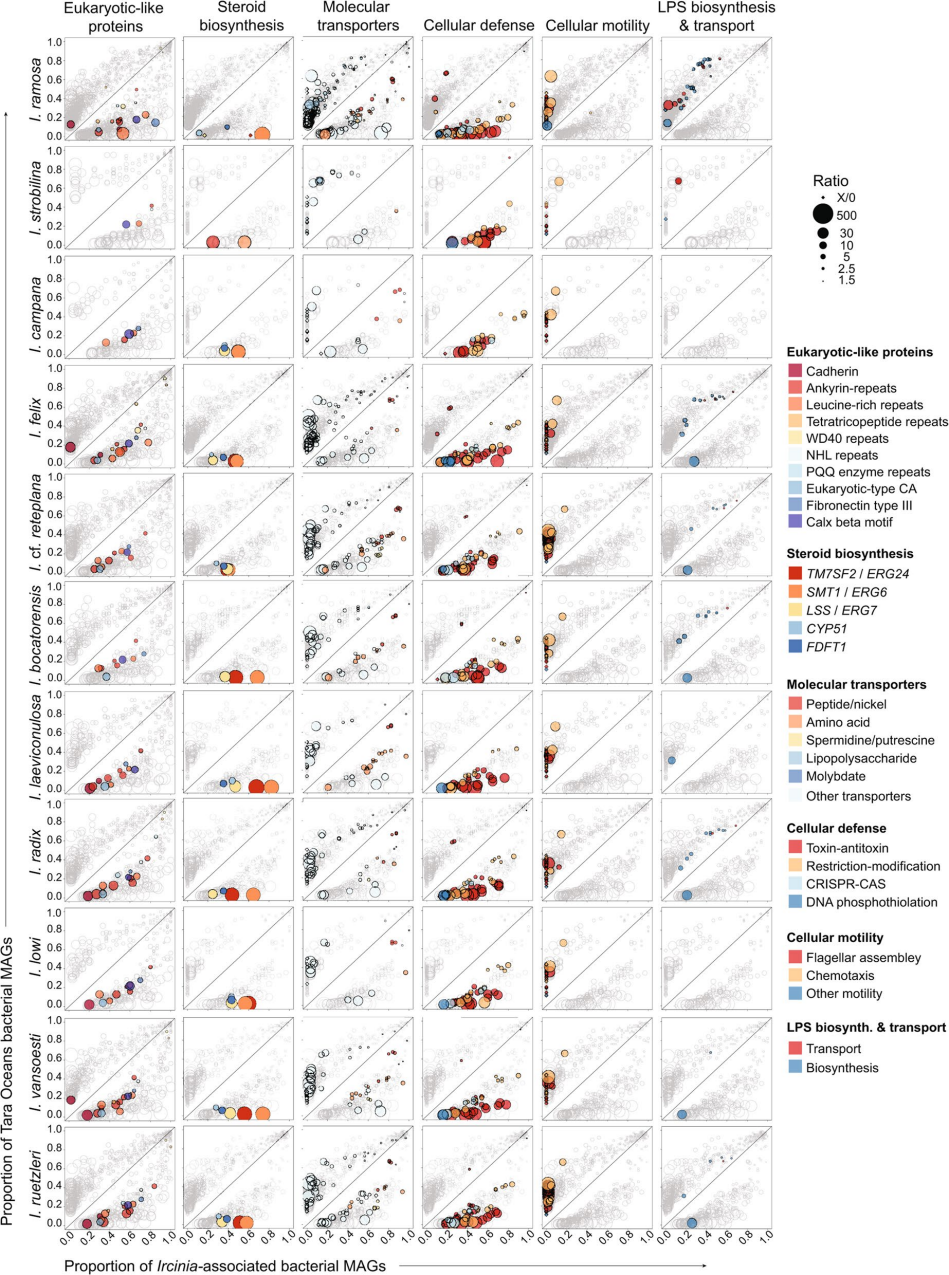
Plots depicting the genes and domains that are enriched or depleted in tropical *Ircinia* spp. Each circle corresponds to a gene that was determined as being differentially abundant in either MAGs sourced from the microbiomes of *Ircinia* or Tara Oceans pelagic seawater samples in terms of normalized average gene copy number. The size of each circle is scaled to the log_10_-transformed ratio of the gene calculated as (avg. copy number in *Ircinia* MAGs)/(avg. copy number in Tara Oceans MAGs) for genes under the *y* = *x* diagonal and (avg. copy number in Tara Oceans MAGs)/(avg. copy number in *Ircinia* MAGs) for genes above the *y* = *x* diagonal. The ELP graph was constructed from the Interproscan annotations; all others were constructed using the KO annotations produced using EnrichM

The three major categories of genes that have been found to be enriched in other sponge-symbiont metagenomes and were investigated here are ELPs, cellular defense, and molecular transporters [20, 53–65]. *Ircinia* MAGs were characterized by a prevalence and diversity of ELPs (Fig. 2, Additional file 3: Fig. S3, Additional file 8: Table S3). In addition to the ankyrin-rich repeats (*ANK*) and *WD40* domains that were previously reported in *I. ramosa* [16], multiple additional classes of ELPs that are enriched in the microbiomes of other host sponge species [20, 53–58, 60, 61] were enriched in *Ircinia*: leucine-rice repeats (*LRR*), fibronectin type III domain (*fn3*), cadherin domains (*CAD*), tetratricopeptide repeats (*TPR*), pyrrolo-quinoline quinone domains (*PQQ*), Lin-41 sequence repeats (*NHL*), and *PD40*. Additionally, two domains that were predicted to be ELPs based on the EffectiveDB database of ELPs [66], Eukaryotic type carbonic anhydrase (PF00194) and Calx-beta motif (PF03160), were enriched across multiple host species and bacterial phyla. Each host species displayed unique signatures of ELP enrichment; for example, enrichment in TPR domains was detected only in *I. radix*, *I. vansoesti*, *I. ruetzleri*, *I. felix*, and *I. ramosa*. In some cases, domains belonging to ELP classes were depleted, as seen in two CADs, PF17803 Cadherin_4 and PF17892 Cadherin_5, which were depleted in multiple host species, and in TPRs, which were depleted as a class in Alphaproteobacteria and PF09976 TPR_21, which was depleted in *I. ramosa*. Actinobacteriota were not enriched for ELPs, consistent with previous analyses of tropical sponge metagenomes [67].

Genes related to cellular defense were also enriched across *Ircinia* metagenomes (Fig. 2, Additional file 3: Fig. S3, Additional file 8: Table S3) including type II T-A systems (*hicA*-*hicB*, *vapC*-*vapB*, *mazF*-*mazE*, *relE*-*relB*, *hipA*-*hipB*, *parE*-*parD*, *chpB*-*chpS*), components of type II and type III restriction-modification (R-M) systems, and CRISPR-CAS-associated genes (*cas1-4*, *csb1-2*). Notable differences across hosts included the enrichment of all species except *I. bocatorensis* and *I. strobilina* for the three subunits of the EcoR124I type I restriction-modification enzyme (*hsdM*, *hsdR*, *hsdS*). *I. felix* and *I. ramosa* were enriched for both components of the type IV R-M system *mcrBC* and three *dnd* sulfur modification genes (*dndB*-*D*). Only *I. ruetzleri* was enriched for the CRISPR system Cascade subunits *casA*/*C*/*D*/*E*. Genes related to defense were also enriched across the five bacterial taxa analyzed independently, although Gammaproteobacteria were not enriched for CRISPR-CAS genes.

A similar signature of enrichment was present among molecular transporters (e.g., ATP-binding cassette transporters) (Fig. 2, Additional file 3: Fig. S3, Additional file 8: Table S3). These included the peptide/nickel transport system comprising the *ddpABCDF* operon (*ddpF*/*D* and *ABC*.*PE.P*/*S*/*P1*), which were enriched in all hosts except *I. strobilina* and were also found to be depleted in Bacteroidota, and amino acid transporters including the branched-chain amino acid transport system genes *livF*/*G*/*H*/*K*/*M*, which were enriched in *I. ramosa*, *I.* cf. *reteplana*, *I. vansoesti*, *I. rutzleri*, and *I. laeviconulosa*. Three of these species (*I. ramosa*, *I.* cf. *reteplana*, and *I. laeviconulosa*) also had strong enrichment patterns for the general l-amino acid transport system genes *aapJ*/*M*/*P*/*Q*. Six species (*I. ramosa*, *I.* cf. *reteplana*, *I. vansoesti*, *I. ruetzleri*, *I. bocatorensis*, and *I. laeviconulosa*) were enriched for the glycine betaine/proline transport system genes *proV*/*W*/*X*. Amino acid transporters were enriched in all five bacterial taxa that were evaluated independently. The putative spermidine/putrescine transport system genes *ABC.SP.A*/*P*/*P1*/*S* were enriched in *I.* cf. *reteplana* and *I. ruetzleri*, with two of these genes enriched in *I. ramosa* (*ABC.SP.A*/*S*) and *I. vansoesti* (*ABC.SP.P*/*S*). *I. ramosa*, *I.* sp. *reteplana*, and *I. ruetzleri* were enriched for sugar/saccharide transport system genes (with the exception of LPS transporters) including the raffinose/stachyose/melibiose transport system (*msmE*/*F*/*G*) and the multiple sugar transport system genes *ABC.MS.P*/*P1*/*S*. *I. ramosa* was also enriched for the two ribose transport system genes *rbsB*/*C* and the simple sugar transport system genes *ABC.SS.A*/*P*. *I. radix* was unique in that only this host was enriched for the molybdate transport system genes *modA*/*B*.

We investigated enrichment patterns for genes whose products are either targeted by host immune systems or are implicated in pathogenicity. We detected depletion of motility genes across tropical *Ircinia* metagenomes (Fig. 2, Additional file 8: Table S3), including the gene that codes for flagellin (*fliC*/*hag*) and 31 other genes involved in flagellar assembly, and 11 chemotaxis-associated genes. Two other categories of genes relevant to microbial pathogenicity that have not yet been subject to scrutiny under a large-scale comparative metagenomic analysis in sponges were depleted in *Ircinia* metagenomes: LPS transport (8 genes) and LPS biosynthesis pathways (19 genes) (Fig. 2, Additional file 8: Table S3). The only exception was *waaQ* (heptosyltransferase III), which was enriched in six of the species (*I.* cf. *reteplana*, *I. vansoesti*, *I. ruetzleri*, *I. felix*, *I. bocatorensis*, and *I. radix*). On the level of individual bacterial phyla, motility genes were only recovered as being completely depleted in Proteobacteria. The bacterial phyla were also depleted for LPS genes, although in Actinobacteriota LPS genes were largely absent and not detected as being enriched nor depleted.

### Enrichment and evolution of steroid biosynthesis genes

**Fig. 3:**
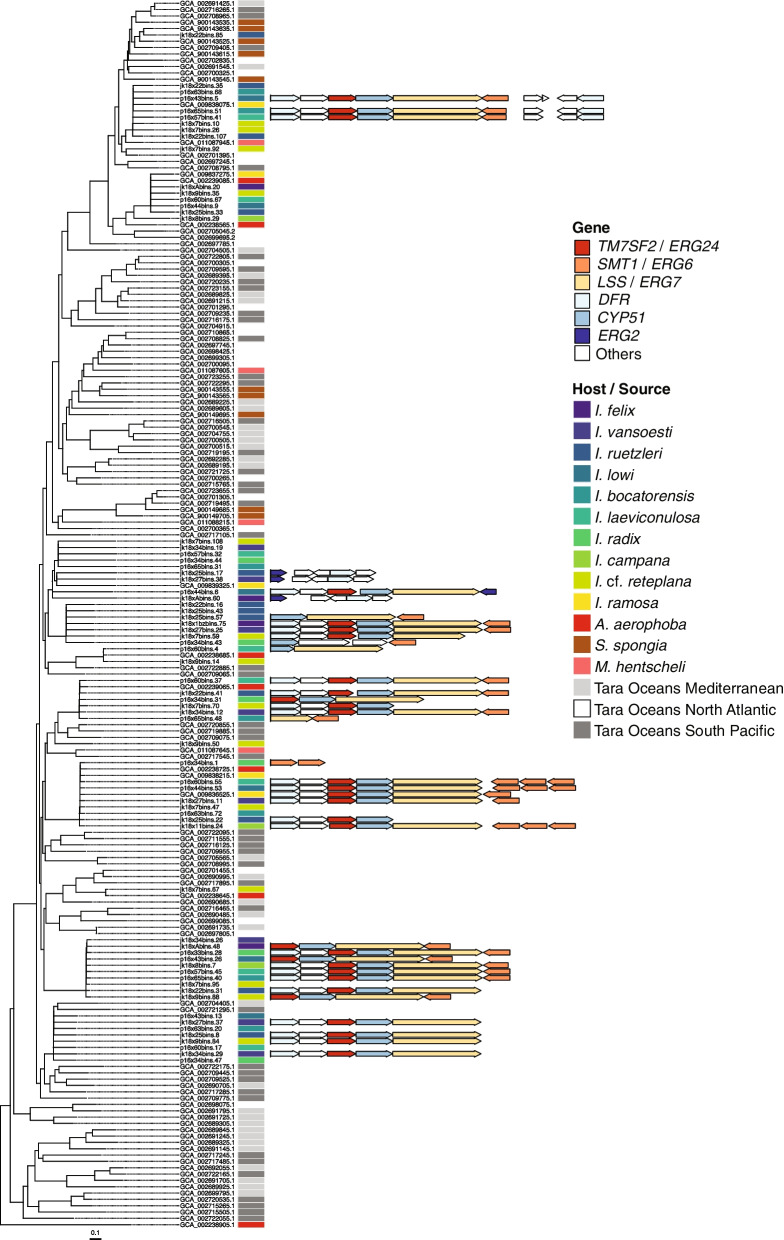
Representative CSGs are depicted on the alphaproteobacterial clade of the GTDB-Tk-inferred phylogeny*.* CSGs are depicted with other genes found in the eukaryotic steroid biosynthesis pathway (PATH:ko00100) that were commonly found flanking the three core genes (*TM7SF2*/*ERG24*, *CYP51*, and *LSS*/*ERG7*) and DFR (dihydroflavonol-4-reductase). “Others” refers to the genes not found in pathway ko00100. If no operon is shown for a given MAG, then a CSG was not detected. Arrows denote the direction of the coding sequence. Genes that overlap with the “steroid biosynthesis” panels of Fig. 2 are colored to match. Tree plotting and annotation were performed with the R packages ggtree [51]

Five genes belonging to the eukaryotic steroid biosynthesis pathway (PATH:ko00100) were discovered to be enriched in tropical *Ircinia* (Fig. 2, Additional file 3: Fig. S3, Additional file 8: Table S3)*.* They were delta 14-sterol reductase (*TM7SF2*/*ERG24*), sterol 24-C-methyltransferase (*SMT1*/*ERG6*), farnesyl-diphosphate farnesyltransferase (*FDFT1*), lanosterol synthase (*LSS*/*ERG7*), and sterol 14alpha-demethylase (*CYP51*) (Fig. 2). These genes often occurred scattered around the bacterial chromosomes or in doublets, as with *TM7SF2*/*ERG24* and *FDFT1*, but three genes regularly clustered together to form an operon: *TM7SF2*/*ERG24*, *CYP51*, and *LSS*/*ERG7* (Fig. 3, Additional file 4: Fig. S4). These clusters of steroid genes (hereafter CSGs) were present in 115 of the 537 MAGs dereplicated within species and were found in 11 classes spanning 10 phyla (Acidimicrobiia (Actinobacteriota), Acidobacteriae (Acidobacteriota), Alphaproteobacteria (Proteobacteria), Binatia (Binatota), Gammaproteobacteria (Proteobacteria), Dehalococcoidia (Chloroflexota), Gemmatimonadetes (Gemmatimonadota), Nitrospiria (Nitrospirota), Rhodothermia (Bacteroidota), UBA8248 (UBA8248), Verrucomicrobiae (Verrucomicrobiota)) and in all 11 *Ircinia* species (Fig. 1, Additional file 4: Fig. S4). Despite being taxonomically widespread, the CSGs were conserved in length (mean length 5910 bp ± 74 bp [1 s.d.], min = 5293 bp, max = 6059) (Additional file 10: Data File S1). The CSGs were usually flanked by dihydroflavonol-4-reductase (*DFR*) and a hypothetical protein (in that order) at the 5′ end oriented on the same strand as the three core genes and by either *SMT1*/*ERG6* or *FDFT1* at the 3′ end, which were coded on the opposite strand (Fig. 3, Additional file 4: Fig. S4).

Given the novelty of the CSGs, we decided to check publicly available sequences for homologous features. MAGs of three other non-*Ircinia* species (*Aplysina aerophoba*, 28 of 41 initial MAGs passing QC and dereplication; *Spongia spongia*, 10 of 10 MAGs; and *Mycale hentscheli*, 13 of 35 MAGs) were checked for the presence of CSGs, which were found in only two MAGs of *A. aerophoba*, GCA_002239025.1 (Chloroflexota) and GCA_002238765.1 (Nitrospirota), and none in the other non-*Ircinia* hosts or Tara Oceans MAGs (Additional file 4: Fig. S4). A homology search using BLASTn that queried representative CSGs from each class and host species against the NCBI nr database yielded the same best hit across queries (mean query cover = 73% ± 32% [1 s.d.], mean *E*-value = 5E−144 ± 1.7E−143, mean percent identity = 74% ± 4%), a CSG from a gammaproteobacterial symbiont of another sponge genus, *Melophlus*, (NCBI accession MT026193.1) (Additional file 9: Table S4) that codes for a low-abundance sarasinoside [68]. In all searches, the next best significant hits had low query covers of (typically less than 5%) and hence were unlikely to constitute homologous CSGs.

The lengths of the *CYP51*, *LSS*/*ERG7*, and *TM7SF2*/*ERG24* codon-aware multiple sequence nucleotide alignments were 1698, 3765, and 1167 positions, respectively, with 12.4%, 21.5%, and 24.3% conserved sites. GTR+I+G [69] was identified by AIC [70] as the model best-fitting nucleotide evolution for all three alignments, calculated in jModelTest2. Bayesian phylogenetic tree inference using the predicted coding sequences for each of the three core genes recovered trees that were not consistent with the genome-wide phylogeny inferred by GTDB-Tk and provided evidence for horizontal gene transfer (HGT) (Additional file 5: Fig. S5). For each gene, clades were typically represented by sequences from only one bacterial class. However, in many cases, the sequences from a given bacterial class were distributed over multiple clades that shared common ancestors with sequences from different bacterial classes before meeting the common ancestor from which all the sequences from the same class were descended. Additionally, the *CYP51* tree was divided into two major clades, each of which contained sequences from Rhodothermia, Gammaproteobacteria, Alphaproteobacteria, and Dehalococcoidia (Additional file 5: Fig. S5A).

**Fig. 4:**
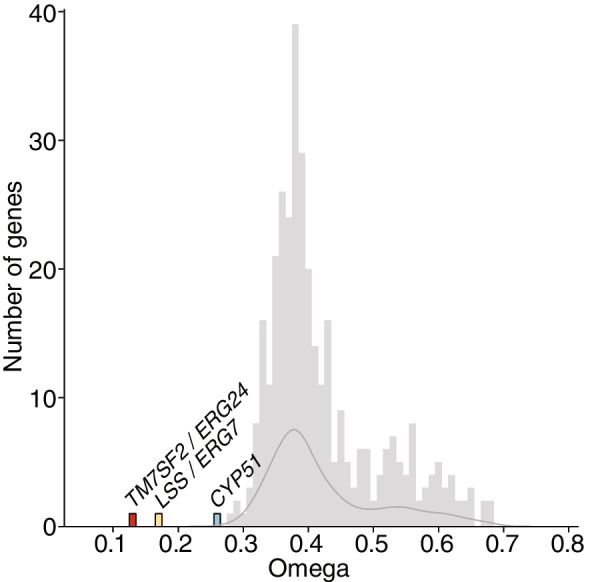
Histogram of omega values for genes with tree-wide average bootstrap scores > 70%. Gray bars correspond to non-steroid biosynthesis genes. The dark gray line marks the density distribution of omega values calculated for non-steroid genes

**Fig. 5:**
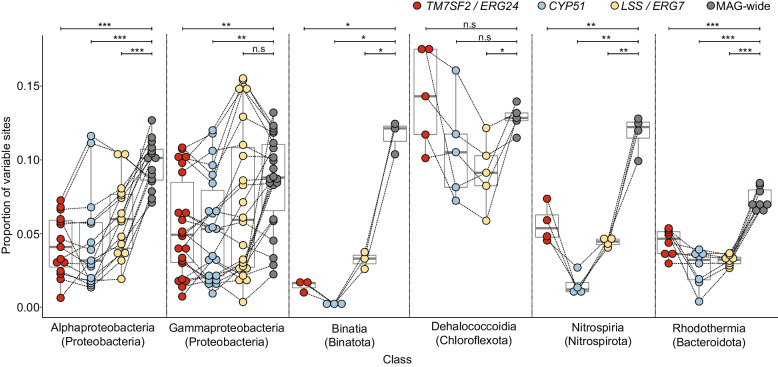
Plots depicting the proportions of nucleotide variability in CSGs compared to MAG-wide values for bacterial classes that had at least three MAGs with CSGs. The lines link the values within each MAG. FDR-corrected *p*-values are reported (*p < 0.05, **p < 0.01, and ***p < 0.001) for pairwise permutational *t*-tests each run for 10,000 iterations comparing the proportion of variable sites within each gene to the genome-wide proportion of variable sites that fall within coding regions. N.s., not significant. The CSG genes are positioned in the order in which they appear in the CSGs

Variant analysis was performed using a dataset of MAGs dereplicated across species by comparing the omega value of each gene relative to the omega values of genes not contained in the CSGs and by analyzing the proportion of variable sites within each gene relative to the MAG-wide average for protein-coding regions. The dereplication scheme resulted in 284 Caribbean *Ircinia* MAGs (mean MAGs per host species = 28.4 ± 15.2 [1 s.d.]), 59 of which (20.8%) had CSGs (Additional file 6: Table S1). A total of 344 non-steroid genes were present in the omega dataset after quality filtering based on tree-wide average ultrafast bootstrap values. The omega values were substantially lower for the three genes contained in the CSGs (*TM7SF2*/*ERG24* omega = 0.13, *LSS*/*ERG7* omega = 0.17, *CYP51* omega = 0.26) (Fig. 4). This trend held in the pre-ultrafast bootstrap filter dataset of 1000 non-steroid genes. The three core CSG genes also generally had significantly lower rates of variability relative to MAG-wide rates across classes (Fig. 5). However, the proportion of variable sites differed across the CSGs by class. For example, in Binatia and Nitrospiria *CYP51* had the lowest rate of variability relative to *TM7SF2*/*ERG24* and *LSS*/*ERG7*, whereas in Dehalococcoidia *LSS/ERG7* had the lowest rate of variability relative to *TM7SF2*/*ERG24* and *CYP51*.

## Discussion

The density and sheer taxonomic richness of tropical *Ircinia* microbiomes, combined with the many ecologically important metabolic processes that they drive such as biogeochemical cycling [12, 13] and photosynthesis [10, 71], render this genus an important study system for our understanding of tropical benthic ecology and host-microbial coevolution. To this end, our results illuminate trends of genome evolution of bacterial symbionts of *Ircinia* and provide insights into how highly complex microbiomes exist within this genus. Combined with prior research investigating genome evolution in *Ircinia* hosts [4], the current study helps complete our view of host-microbial evolution in sponges.

A yet unanswered question in sponge biology is how hosts can tolerate dense and diverse microbiomes. Four categories directly relevant to pathogenicity that were investigated were LPS biosynthesis, LPS transport, motility (including the flagellin gene *fliC*/*hag* and other flagellar assembly proteins), and chemotaxis, which were depleted in *Ircinia* microbiomes. Motility and chemotaxis genes have been found to be depleted in the metagenomes of *Lamellodysidea herbaceae* (particularly flagellar biosynthesis genes) [64], *Petrosia ficiformis*, *Sarcotragus foetidus*, and *Aplysina aerophoba* [63], in a sulfur-oxidizing gammaproteobacterial symbiont of *Suberites* sp. [72] and in a *Rhodospirillaceae* symbiont of *Spongia officinalis* [57]. Prior research also found that genes responsible for the biosynthesis of l-rhamnose, a component of the O-antigen of LPS, were missing from the genomes of four “*Ca.* S. spongiarum” strains, each inhabiting a different host sponge species: *Theonella swinhoei*, *Ircinia variabilis*, *A. aerophoba*, and *Carteriospongia foliascens* [17]. The modification to the LPS of “*Ca.* S. spongiarum” is speculated to prevent recognition and removal by host archaeocytes, a hypothesis supported by the finding that another cyanobacterial symbiont, “*Ca.* S. feldmannii,” which is intracellular and thus not exposed to archaeocytes, does in fact have conventional O-antigen based on the presence l-rhamnose biosynthesis genes [18]. While it is not known whether the present MAGs represent intra or extracellular bacteria, given that LPS and flagellar components elicit immune responses that can be damaging for both the host and symbionts [73–76] and that LPS-induced pathways are present in sponges [77–79] and are under selection in Caribbean *Ircinia* [4], the hypothesis could be extended to state that loss of LPS and motility genes are common features among bacterial symbionts of *Ircinia* and non-*Ircinia* hosts and represent another common strategy to avoid inciting the host’s immune system in addition to ELP enrichment, another widespread feature of sponge symbionts [20, 53–58, 60, 61].

The enrichment patterns of three categories of transporters (amino acid, spermidine/putrescine, sugar/saccharide) provide further indications into how the symbiotic microbes have evolved nutritional strategies within *Ircinia* and suggest that transporters are an adaption to sponges in general [20, 53–65]. Like other members of class Demospongiae, *Ircinia* possess high concentrations of free amino acids within their tissues [80], which could not only provide abundant sources of amino acids for protein biosynthesis but also the source molecules for the breakdown products spermidine and putrescine, two polyamines that are essential for cell growth [81] and which perform protective functions for host-associated prokaryotes [82]. Additionally, the enrichment of sugar transporters suggests that, like the symbionts of other sponges [83, 84], the microbiomes of *Ircinia* are active contributors in microbial processes that drive dissolved organic matter (DOM) turnover in tropical benthic environments.

*Ircinia* are known for their production of antibiotics (reviewed in [85]). The enrichment of genes within the peptide/nickel transporter category comprising the *ddpABCDF* operon could be an evolutionary response to the presence of such chemicals, as it is involved in cell wall restructuring that confers vancomycin resistance [86]. Curiously, no genes belonging to the biosynthesis of vancomycin group antibiotics pathway (PATH:ko01055) were enriched in sponge metagenomes, although several of these genes were indeed present in the MAGs of *Ircinia* symbionts.

Sterols are rare occurrences in bacteria, although they have been found to be produced by a handful of free-living and host-associated bacteria [68, 87]. The biogenic source of these compounds in sponges is still debated, especially for sterols with 24-C side-chain modifications such as 24-isopropylcholesterol, which is used as a biomarker to date sponge fossils to the Neoproterozoic Era [88]. *SMT1/ERG6*, a gene that can perform this modification, was found to be enriched in *Ircinia* bacterial MAGs, and furthermore, *SMT* copies derived from bacterial symbionts of *A. aerophoba* were recently demonstrated in vitro to perform sequential methylations at the 24-C position that produce the 24-isopropyl side-chain [89]. However, assignment of 24-isopropylcholesterol synthesis to symbiotic bacteria will require a more in-depth pathway characterization of sterol biosynthesis, and comparisons of molecular clock dating estimates of host and symbiont-derived *SMT1/ERG6* copies could help investigate whether the genesis of bacterial copies are old enough to have contributed to 24-isopropylcholesterol in Neoproterozoic sponge fossils, as has already been done to date the earliest algal *SMT* gene duplication to the later Phanerozoic Eon [90].

Sterol production has been either observed or predicted based on genetic data for eight of the ten bacterial phyla containing CSGs in *Ircinia* [87, 91–96] but, to our knowledge, has never been predicted in Binatota or UBA8248. The CSGs are similar in gene content and are conserved in synteny relative to described putative bacterial steroid formation and modification operons [91], with the C-24 modification gene (*SMT1/ERG6*) co-occurring with the O_2_-dependent steps of cyclization (*LSS*/*ERG7*), C-14 demethylation (*CYP51* and *TM7SF2*/*ERG24*) and, in several of the CSGs, the modification of desaturation step (*ERG2*). Our pipeline did not annotate *SdmA* and *Sdmb*, the genes involved in the C-4 demethylation step, although these could be annotated as the hypothetical proteins within the CSGs.

In addition to the conservation of organization and gene content within the CSGs, other lines of evidence in our data support the hypothesis that they are functionally important to bacterial symbionts of *Ircinia.* One interpretation of the evidence for HGT and the widespread distribution of taxa possessing the CSGs is that they are a necessity in the environment of the sponge interior, as is the case for antibiotic resistance genes that undergo HGT in pathogens [97–100]. This hypothesis is corroborated by the fact that the CSGs are absent from the Tara Oceans dataset and are otherwise a scarcity in the bacterial domain [87]. Additionally, the low omega values and low levels of nucleotide variance within the CSGs compared to MAG-wide averages suggest that they are undergoing purifying selection and are thus constrained in evolution at the level of the molecular residue [101–103].

A potential caveat of our enrichment analysis should be noted in that some phyla are overrepresented in *Ircinia* microbiomes relative to the Tara Oceans dataset that could be contributing unique signatures of gene content. This was partially captured in our analysis of individual bacterial phyla (and classes, in the case of Proteobacteria), which demonstrated that enrichment trends are not uniform across taxa. Thus, the patterns of genomic enrichment are best interpreted as being produced by a combination of environmental filtering, including selection by the host, and different patterns of genome evolution that are the result of selective regimes inside the sponge.

It should be noted that the sponge MAG dataset has a higher level of geographic resolution than the Tara Oceans MAG dataset. This is due to the fact that the authors of the latter dataset produced MAGs specific to the level of oceanic province [34]. Based on the coordinates of the sampling stations, these MAGs could include genomic content found only in bacteria that occur at higher latitudes and more eastward longitudes in the North Atlantic and South Pacific relative to the sampling locations of our sponge dataset, potentially introducing geographical bias. However, the inclusion of pelagic MAGs derived from a broader geographic range would also be expected to increase the probability that CSGs would be detected, which was not the case. Furthermore, our results are concurrent in several regards with patterns of genome evolution that have been detected in other sponge symbionts such as enrichment of ELPs, genes involved in cellular defense, and molecular transporters [20, 53–64]. Nevertheless, the testing of hypotheses regarding the effects of sponge inhabitation on bacterial genome evolution could be enhanced by future work that uses a free-living bacterial MAG dataset that has a level of geographic resolution matching that of the sponge dataset.

## Conclusion

Tropical *Ircinia* are recognized for their roles in driving ecologically important metabolic processes that impact the coral reef, seagrass, and mangrove environments in which they reside. The current study provides an investigation of microbial genome evolution of *Ircinia* symbionts across multiple host species and has identified aspects of primary and secondary metabolism of tropical *Ircinia* microbiomes, including the putative capacity to synthesize sterols, that support this genus as being central to microbial and chemical diversity in benthic habitats. Our results also highlight the potential for *Ircinia* to buffer their environments against chemical fluctuations in the seawater [13]. In the Caribbean, this buffering capacity could be further supported by the existence of a regional pool of microbes that are shared among the host species and the metabolic redundancy within each species’ microbiome. These ecological roles, combined with the likely presence of undiscovered bioactive secondary compounds in *Ircinia*, underline the importance of protecting this genus and its habitats.

## Supplementary Information

**Additional file 1: Fig. S1.** Phylogenetic depiction of MAG distributions across Caribbean *Ircinia* species and redundancy in primary metabolic modes. The heatmap immediately to the right of the tree indicates the ln-transformed relative abundances of each MAG by host specimen. Species abbreviations accompanying specimen labels at the bottom of the heatmap are as follows: ICam (*I. campana*), IcfR (*I.* cf. *reteplana*), IRad (*I. radix*), ILae (*I*. *laeviconulosa*), IBoc (*I. bocatorensis* B), ILow (*I. lowi*), IVan (*I. vansoesti*), IRue (*I. ruetzleri*), IFel (*I. felix*), IStr (*I. strobilina*). To the right, the presence and absence of genes are annotated that belong to the six autotrophic carbon cycling pathways of prokaryotes (the Wood-Ljungdahl pathway, the dicarboxylate/4-hydroxybutyrate cycle, the 3-hydroxypropionate bicycle, the 3-hydroxypropionate/4-hydroxybutyrate cycle, the reductive citric acid cycle, and the Calvin–Benson–Bassham cycle), nitrogen metabolism (PATH:ko00910), sulfur metabolism (PATH:ko00920), and methane metabolism (PATH:ko00680). Plotting was performed using the GTDB-Tk bacterial phylogeny and ggtree [51].

**Additional file 2: Fig. S2.** Plots depicting prevalence of MAGs associated with Caribbean *Ircinia* by source and region, inferred using the relative abundance matrix produced by CoverM. **A.** Bar chart depicting the percent of MAGs that are found across multiple host species. **B.** Bar chart depicting the regional specificity of MAGs. **C.** PCA of taxonomic community compositions of each host species’ microbiome.

**Additional file 3: Fig. S3.** Plots depicting genes and domains that that are enriched or depleted in tropical *Ircinia* spp. at the level of bacterial phylum. Plotting scheme follows that of Fig. 2.

**Additional file 4: Fig. S4.** CSGs plotted annotated on bacterial phylogeny comprising sponge-derived (*Ircinia* and non-*Ircinia*) and Tara Oceans MAGs, following tree construction and plotting scheme as outlined for Fig. 3.

**Additional file 5: Fig. S5.** Midpoint-rooted phylogenetic trees for three genes found in CSGs, inferred in MrBayes. Nodes with less than 50% posterior probabilities are collapsed.

**Additional file 6: Table S1**. Taxonomy, statistics, source metadata, and CSG occurrence in MAGs that passed QC and dereplication steps and were used in the final analyses. The archaeal MAGs and the *Trichodesmium* MAG that did not meet the completeness threshold are included.

**Additional file 7: Table S2.** Table of relative abundances of *Synechococcus* MAGs in *Ircinia*, inferred via CoverM.

**Additional file 8: Table S3.** Table of genes and domains that are significantly enriched or depleted in *Ircinia*, and which are plotted in Fig. 2.

**Additional file 9: Table S4.** The top BLASTn hits for representative CSGs.

**Additional file 10: Data File S1.** Fasta-formatted CSGs found in bacterial symbionts of *Ircinia*.

## Data Availability

The raw sequencing read and assembled MAG datasets supporting the conclusions of this article are available in NCBI’s GenBank repository under BioProject ID PRJNA768976 [https://www.ncbi.nlm.nih.gov/bioproject/?term=PRJNA768976]. Physical vouchers of the sponge specimens used in this study are deposited with the Smithsonian National Museum of Natural History under the following accession numbers: *I. campana*: 1641986 and 1641983; *I.* cf. *reteplana*: 1641982 and 1641984; *I. felix*: 1642022 and 1641991; *I. vansoesti* (*I.* sp. “BZ1”): 1642005 and 1642012; *I. ruetzleri* (*I.* sp. “BZ2”): 1642000 and 1642003; *I. lowi* (*I.* sp. “Encrusting”): 1582269 and 1582270; *I. laeviconulosa* (*I.* sp. “Massive A green”): 1582283 and 1582286; *I. radix* (*I.* sp. “Massive A pink”): 1582259 and 1582260; *I. bocatorensis* (*I.* sp. “Massive B”): 1582289 and 1582291; and *I. strobilina*: 1642018 and 1642021. The code required to reproduce the analyses can be found under https://github.com/jbkelly8686/Ircinia-Metagenomics and https://github.com/davidecarlson/Snakemake-bcftools .
