# Correction: Novel trends of genome evolution in highly complex tropical sponge microbiomes

**DOI:** 10.1186/s40168-022-01393-x

**Published:** 2022-10-24

**Authors:** Joseph B. Kelly, David E. Carlson, Jun Siong Low, Robert W. Thacker

**Affiliations:** 1 Aquatic Ecology and Evolution, Limnological Institute University Konstanz, Konstanz, Germany; 2 Department of Ecology and Evolution, Stony Brook University, Stony Brook, NY USA; 3 Institute of Microbiology,ETH Zürich, Zürich, Switzerland; 4 Institute for Research in Biomedicine, Università della Svizzera Italiana, Bellinzona, Switzerland; 5 Department of Immunobiology, Yale University School of Medicine, New Haven, CT USA; 6 Smithsonian Tropical Research Institute, Box 0843-03092, Balboa, Panama City, Republic of Panama

**Correction: Microbiome 10, 164 (2022)**

**https://doi.org/10.1186/s40168-022-01359-z**

Following the publication of the original article [1], the author reported that the affiliations were incorrectly assigned.

This has been corrected and the original article has been updated.
