# Supplementary material for: Novel trends of genome evolution in highly complex tropical sponge microbiomes
Source: Microbiome. 2022 Oct 4;10:164. doi: 10.1186/s40168-022-01359-z (PMC9531527; doi:10.1186/s40168-022-01359-z)
Supplement: Supplementary file 2 — Additional file 1: Fig. S1. Phylogenetic depiction of MAG distributions across Caribbean Ircinia species and redundancy in primary metabolic modes. The heatmap immediately to the right of the tree indicates the ln-transformed relative abundances of each MAG by host specimen. Species abbreviations accompanying specimen labels at the bottom of the heatmap are as follows: ICam (I. campana), IcfR (I. cf. reteplana), IRad (I. radix), ILae (I. laeviconulosa), IBoc (I. bocatorensis B), ILow (I. lowi), IVan (I. vansoesti), IRue (I. ruetzleri), IFel (I. felix), IStr (I. strobilina). To the right, the presence and absence of genes are annotated that belong to the six autotrophic carbon cycling pathways of prokaryotes (the Wood-Ljungdahl pathway, the dicarboxylate/4-hydroxybutyrate cycle, the 3-hydroxypropionate bicycle, the 3-hydroxypropionate/4-hydroxybutyrate cycle, the reductive citric acid cycle, and the Calvin–Benson–Bassham cycle), nitrogen metabolism (PATH:ko00910), sulfur metabolism (PATH:ko00920), and methane metabolism (PATH:ko00680). Plotting was performed using the GTDB-Tk bacterial phylogeny and ggtree [51]. [file 40168_2022_1359_MOESM1_ESM.pdf]

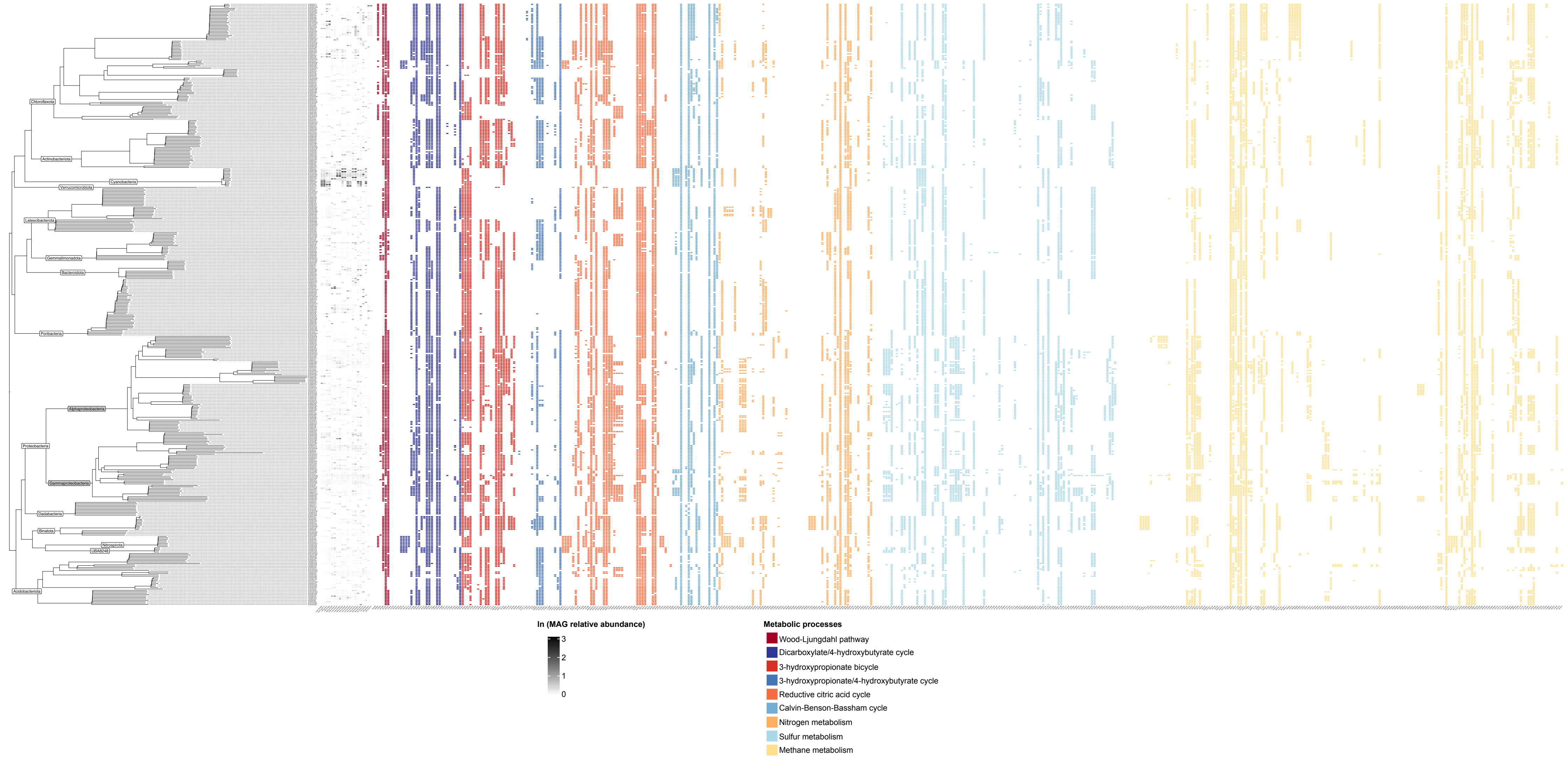
Chloroflexota
Actinobacteriota
Cyanobacteria
Verrucomicrobiota
Latescibacterota
Gemmatimonadota
Bacteroidota
Poribacteria
Alphaproteobacteria
Proteobacteria
Gammaproteobacteria
Dadabacteria
Binatota
Nitrospirota
UBA8248
Acidobacteriota
ln (MAG relative abundance)
3
2
1
0
Metabolic processes
Wood-Ljungdahl pathway
Dicarboxylate/4-hydroxybutyrate cycle
3-hydroxypropionate bicycle
3-hydroxypropionate/4-hydroxybutyrate cycle
Reductive citric acid cycle
Calvin-Benson-Bassham cycle
Nitrogen metabolism
Sulfur metabolism
Methane metabolism
