# Supplementary figures and images for: Novel trends of genome evolution in highly complex tropical sponge microbiomes
Source: Microbiome. 2022 Oct 4;10:164. doi: 10.1186/s40168-022-01359-z (PMC9531527; doi:10.1186/s40168-022-01359-z)

**C**

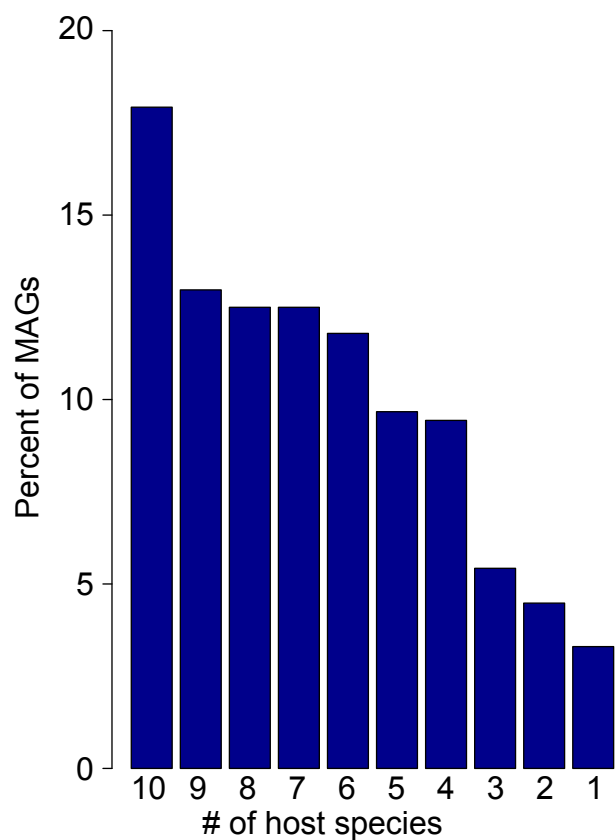

# B

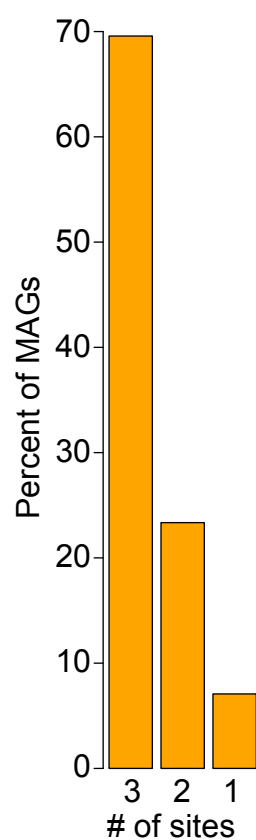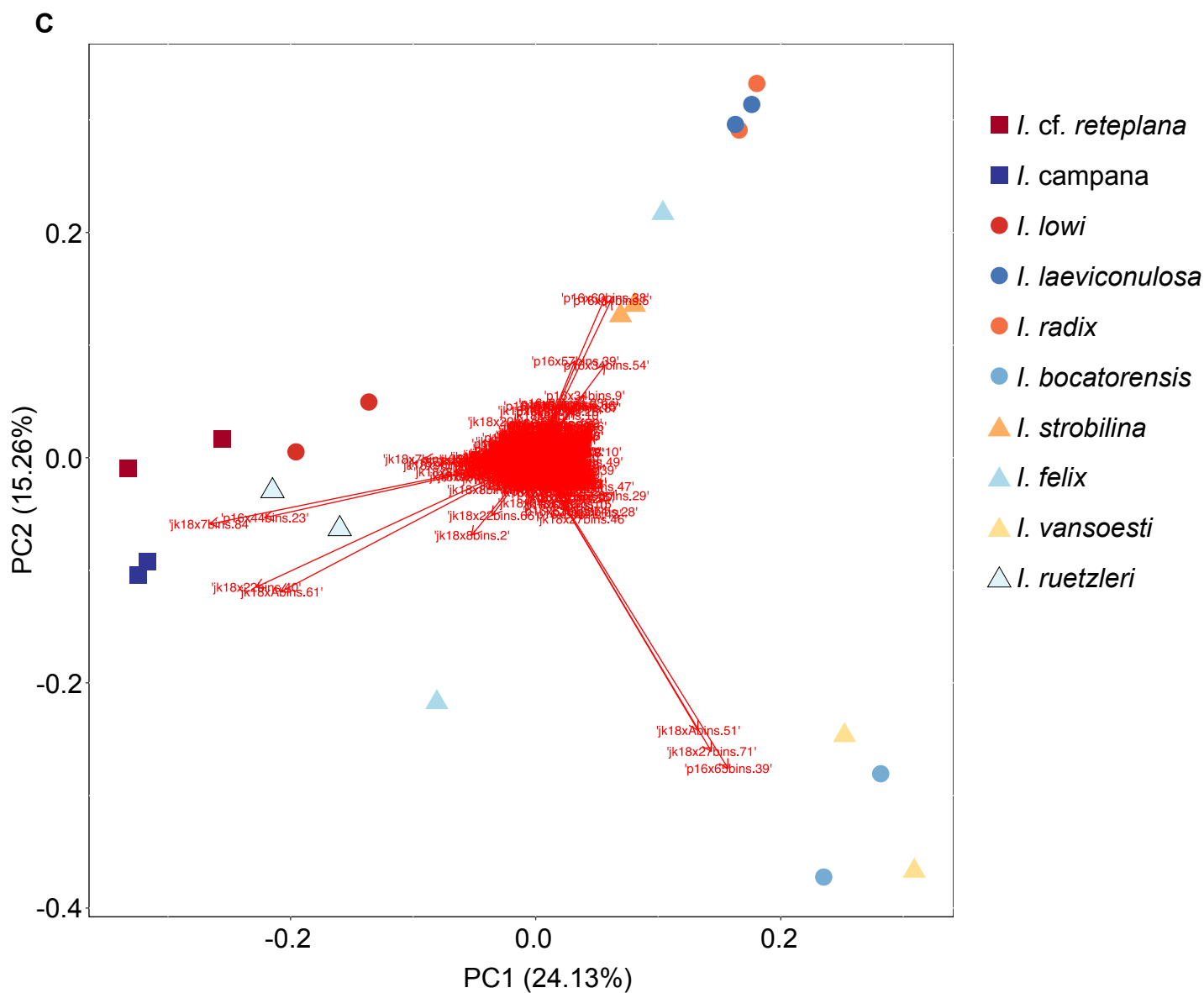

Supplement: Supplementary file 3 — Additional file 2: Fig. S2. Plots depicting prevalence of MAGs associated with Caribbean Ircinia by source and region, inferred using the relative abundance matrix produced by CoverM. A. Bar chart depicting the percent of MAGs that are found across multiple host species. B. Bar chart depicting the regional specificity of MAGs. C. PCA of taxonomic community compositions of each host species’ microbiome. [file 40168_2022_1359_MOESM2_ESM.pdf]

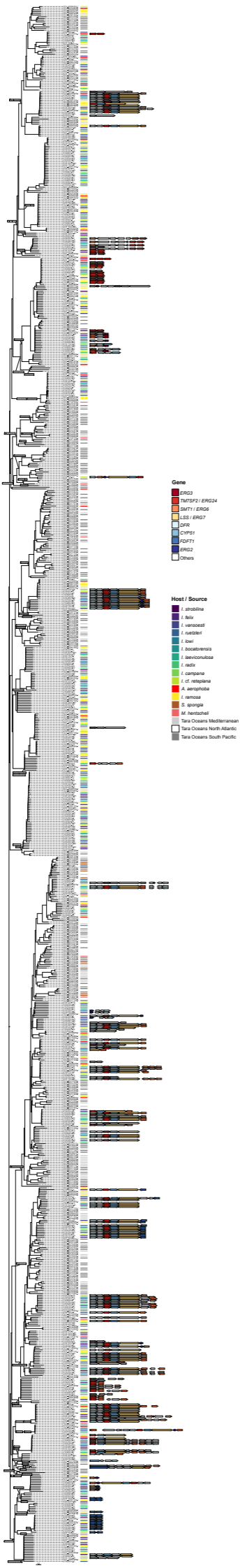

Supplement: Supplementary file 5 — Additional file 4: Fig. S4. CSGs plotted annotated on bacterial phylogeny comprising sponge-derived (Ircinia and non-Ircinia) and Tara Oceans MAGs, following tree construction and plotting scheme as outlined for Fig. 3. [file 40168_2022_1359_MOESM4_ESM.pdf]
