# Supplementary material for: Novel trends of genome evolution in highly complex tropical sponge microbiomes
Source: Microbiome. 2022 Oct 4;10:164. doi: 10.1186/s40168-022-01359-z (PMC9531527; doi:10.1186/s40168-022-01359-z)
Supplement: Supplementary file 4 — Additional file 3: Fig. S3. Plots depicting genes and domains that that are enriched or depleted in tropical Ircinia spp. at the level of bacterial phylum. Plotting scheme follows that of Fig. 2. [file 40168_2022_1359_MOESM3_ESM.pdf]

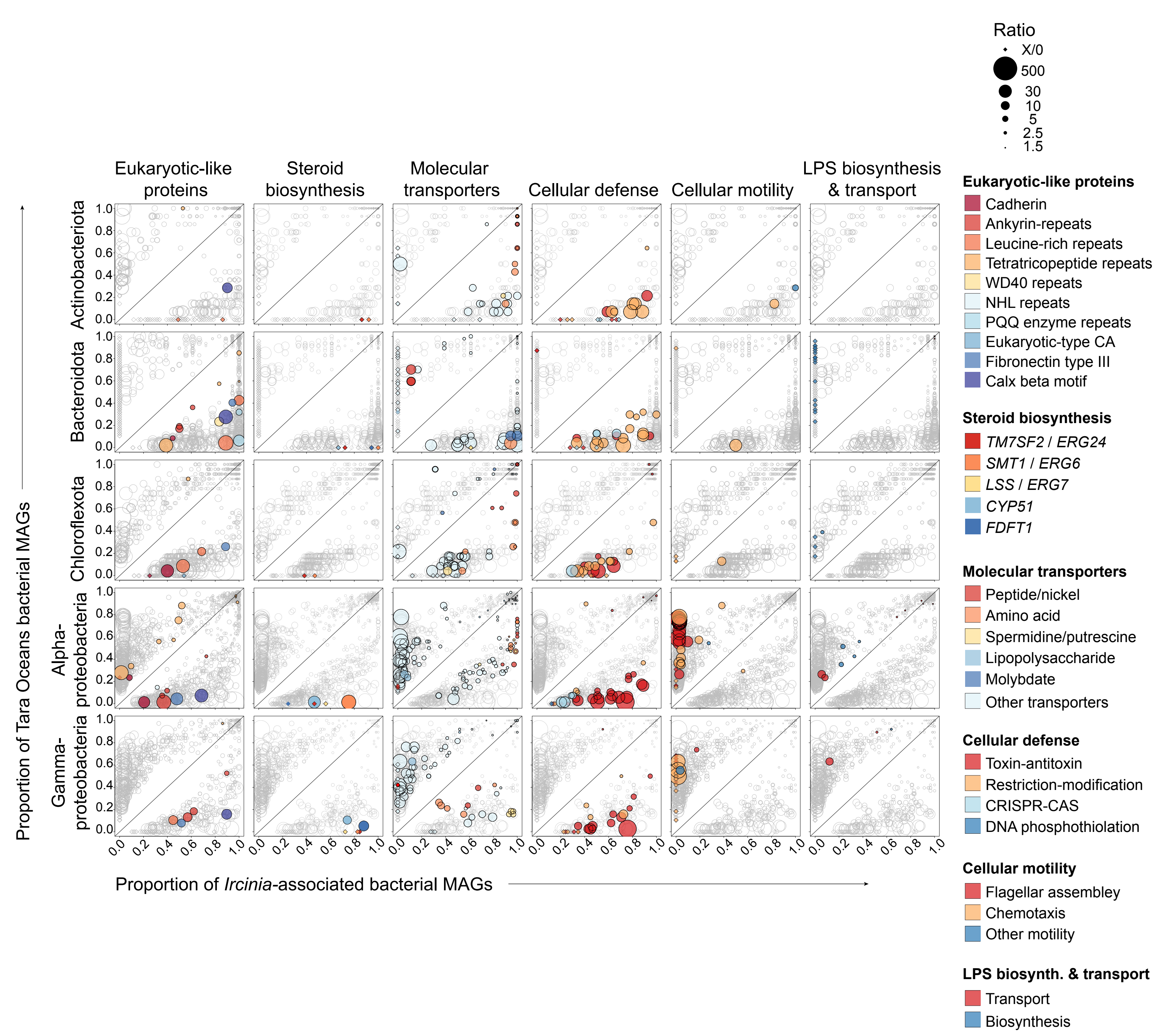

Ratio
X/0
500
30
10
5
2.5
1.5
Eukaryotic-like proteins
Steroid biosynthesis
Molecular transporters
Cellular defense
Cellular motility
LPS biosynthesis & transport
Actinobacteriota
Bacteroidota
Chloroflexota
Alpha-proteobacteria
Gamma-proteobacteria
0.0
0.2
0.4
0.6
0.8
1.0
Proportion of Tara Oceans bacterial MAGs
Proportion of *Ircinia*-associated bacterial MAGs
Eukaryotic-like proteins
Cadherin
Ankyrin-repeats
Leucine-rich repeats
Tetratricopeptide repeats
WD40 repeats
NHL repeats
PQQ enzyme repeats
Eukaryotic-type CA
Fibronectin type III
Calx beta motif
Steroid biosynthesis
TM7SF2 / ERG24
SMT1 / ERG6
LSS / ERG7
CYP51
FDFT1
Molecular transporters
Peptide/nickel
Amino acid
Spermidine/putrescine
Lipopolysaccharide
Molybdate
Other transporters
Cellular defense
Toxin-antitoxin
Restriction-modification
CRISPR-CAS
DNA phosphothiolation
Cellular motility
Flagellar assembley
Chemotaxis
Other motility
LPS biosynth. & transport
Transport
Biosynthesis
