# Supplementary material for: Novel trends of genome evolution in highly complex tropical sponge microbiomes
Source: Microbiome. 2022 Oct 4;10:164. doi: 10.1186/s40168-022-01359-z (PMC9531527; doi:10.1186/s40168-022-01359-z)
Supplement: Supplementary file 6 — Additional file 5: Fig. S5. Midpoint-rooted phylogenetic trees for three genes found in CSGs, inferred in MrBayes. Nodes with less than 50% posterior probabilities are collapsed. [file 40168_2022_1359_MOESM5_ESM.pdf]

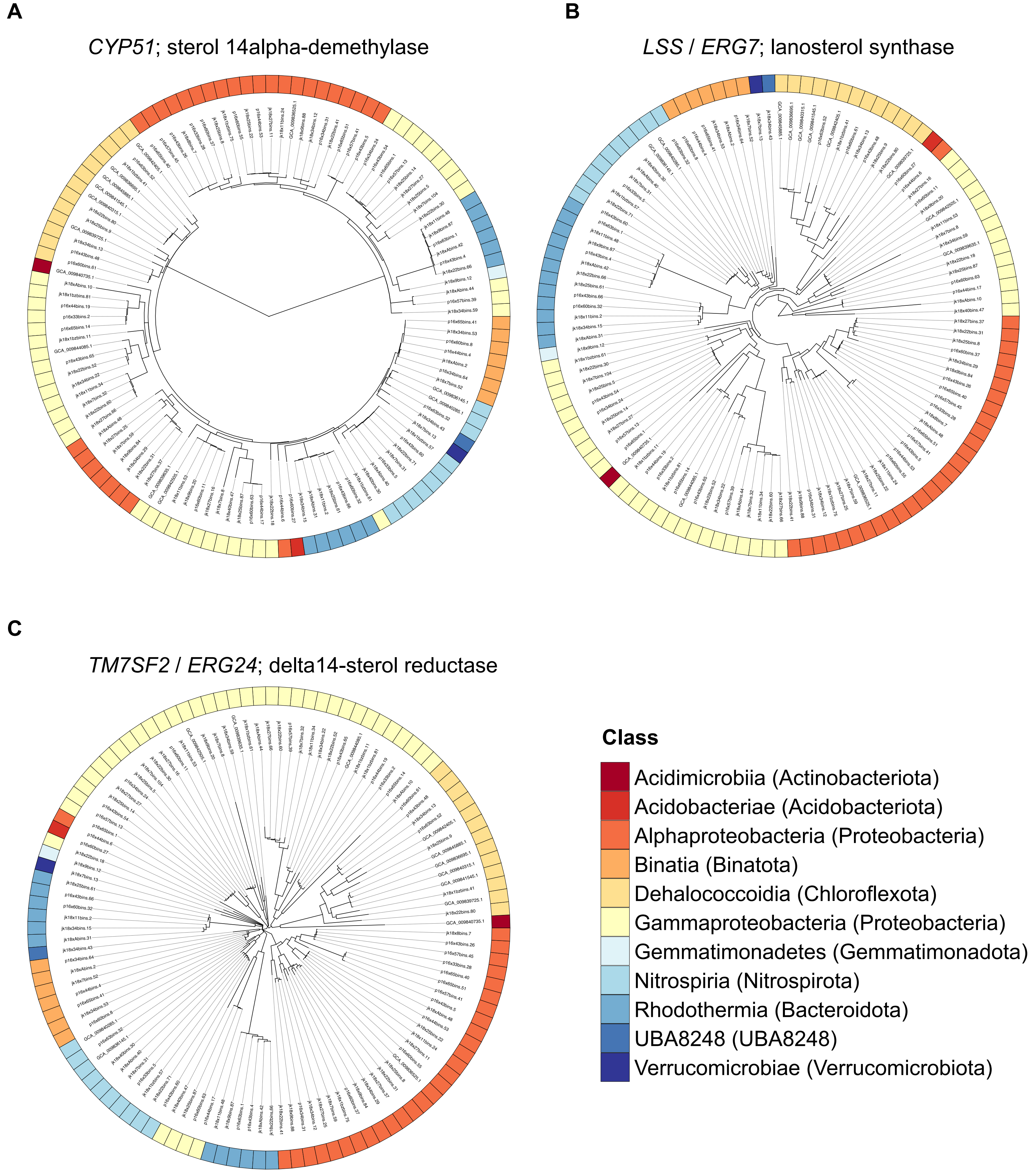
A
CYP51; sterol 14alpha-demethylase
B
LSS / ERG7; lanosterol synthase
C
TM7SF2 / ERG24; delta14-sterol reductase
Class
Acidimicrobiia (Actinobacteriota)
Acidobacteriae (Acidobacteriota)
Alphaproteobacteria (Proteobacteria)
Binatia (Binatota)
Dehalococcoidia (Chloroflexota)
Gammaproteobacteria (Proteobacteria)
Gemmatimonadetes (Gemmatimonadota)
Nitrospiria (Nitrospirota)
Rhodothermia (Bacteroidota)
UBA8248 (UBA8248)
Verrucomicrobiae (Verrucomicrobiota)
